# Development of Genome-Wide Intron Length Polymorphism (ILP) Markers in Tea Plant (*Camellia sinensis*) and Related Applications for Genetics Research

**DOI:** 10.3390/ijms25063241

**Published:** 2024-03-13

**Authors:** Yuan Shen, Xiaoying He, Feng Zu, Xiaoxia Huang, Shihua Yin, Lifei Wang, Fang Geng, Xiaomao Cheng

**Affiliations:** 1 Southwest Landscape Architecture Engineering Research Center of State Forestry and Grassland Administration, College of Landscape Architecture and Horticulture Sciences, Southwest Forestry University, Kunming 650224, China; 15187871158@163.com (Y.S.); fanggeng20@swfu.edu.cn (F.G.); 2 Industrial Crops Research Institute, Yunnan Academy of Agricultural Sciences, Kunming 650225, Chinazufeng1984@163.com (F.Z.)

**Keywords:** *Camellia tetracocca*, intron length polymorphism (ILP), transferability, genetic diversity, population structure

## Abstract

The market value of tea is largely dependent on the tea species and cultivar. Therefore, it is important to develop efficient molecular markers covering the entire tea genome that can be used for the identification of tea varieties, marker-assisted breeding, and mapping important quantitative trait loci for beneficial traits. In this study, genome-wide molecular markers based on intron length polymorphism (ILP) were developed for tea trees. A total of 479, 1393, and 1342 tea ILP markers were identified using the PCR method in silico from the ‘Shuchazao’ scaffold genome, the chromosome-level genome of ‘Longjing 43’, and the ancient tea DASZ chromosome-level genome, respectively. A total of 230 tea ILP markers were used to amplify six tea tree species. Among these, 213 pairs of primers successfully characterize products in all six species, with 112 primer pairs exhibiting polymorphism. The polymorphism rate of primer pairs increased with the improvement in reference genome assembly quality level. The cross-species transferability analysis of 35 primer pairs of tea ILP markers showed an average amplification rate of 85.17% through 11 species in 6 families, with high transferability in *Camellia reticulata* and tobacco. We also used 40 pairs of tea ILP primers to evaluate the genetic diversity and population structure of *C. tetracocca* with 176 plants from Puan County, Guizhou Province, China. These genome-wide markers will be a valuable resource for genetic diversity analysis, marker-assisted breeding, and variety identification in tea, providing important information for the tea industry.

## 1. Introduction

The tea plant (*C. sinensis* (L.) O. Kuntze) is renowned worldwide as one of the most popular non-alcoholic beverages, providing characteristic secondary metabolites such as catechins, theanine, and caffeine, which offer numerous health benefits for humans [1,2,3]. The tea plant originated in the southwest of China and has since expanded to more than 50 countries [4,5]. The main tea-cultivating countries, including China, India, Japan, and Kenya, have embarked on a significant initiative involving the continuous genetic improvement of tea plants. Over 1200 tea cultivars with distinct traits have been developed and released for cultivation worldwide [6,7]. Based on different genetic backgrounds, cultivated tea plants are categorized into two main varieties: *C. sinensis* var. *sinensis* (CSS) and *C. sinensis* var. *assamica* (CSA) [8]. However, *C. tetracocca*, another cultivated tea plant in Puan, Guizhou Province, is also utilized as a tea breeding material, facilitating distant crosses and gene transfer due to its high cold resistance gene source. It is currently important to make breakthroughs in the breeding of excellent tea varieties. Thus, there is an urgent requirement for a simple and accurate method for the identification of varieties, kinship, and phylogenetic analysis of tea plants [6].

The genome size of the tea plant is approximately 3 Gbp, and it exhibits a high level of genetic diversity [9]. The early classification and identification of tea plants were primarily based on morphological characteristics. However, due to a limited understanding of the genetic information in the tea genome, traditional breeding methods have been somewhat blind and inefficient [10]. This limitation poses challenges in meeting the evolving market demands for diverse tea cultivars [11]. Given that the market value of tea is significantly influenced by the tea plant species or cultivars used, it becomes crucial to develop markers capable of distinguishing between different species and their cultivars, regardless of their geographic origin and environmental conditions. While various morphological markers have been developed and utilized through tea species and varieties [12,13], these are often susceptible to environmental influences, leading to difficulties in discriminating cultivars or hybrids derived from genetically related species. Consequently, there is a burgeoning interest in the development of effective DNA-based markers. Such markers not only aid in genetic identification but also present an opportunity to leverage genetic diversity within the tea plant for genetic conservation efforts and targeted breeding.

With the advancement of biotechnology, genetic molecular marker technology has progressively become mainstream, assuming an increasingly pivotal role in genetic diversity analysis, genetic map construction, gene identification, and genetic breeding [14]. Numerous molecular markers for tea plants have been developed. Leveraging recently published genome assemblies [8] and the tea genome database [15], a multitude of locus-specific microsatellite markers have been established [16,17]. Intron length polymorphism (ILP) stands out as a novel molecular marker based on sequenced genomes, possessing advantages such as abundance, high polymorphism, greater reliability, and high cross-transferability through related species when compared to other markers [18]. Presently, two primary approaches are employed for ILP marker development [19,20]. The first approach is rooted in highly conserved intron positions and exon–intron structures among species. ILP markers are created by identifying intron positions through EST/cDNA sequence comparison with homologous sequences of related model plants. A comprehensive effort resulted in the identification of 57,658 potential ILP markers from 59 plant species, and a web-based database of ILP markers was established [19]. The second approach involves species with reference genomes, where the initial step is locating the position of genomic introns [20]. Subsequently, primers are designed based on exons on both sides of the introns, and amplification is achieved through polymerase chain reaction (PCR) to detect ILP. Numerous ILP markers have been successfully applied in diverse crops such as rice [21], foxtail millet [22], onion [23], and carrot [24].

Molecular markers have proven to be invaluable for the accurate assessment of tea genetic resources, contributing significantly to the enrichment of genetic diversity and facilitating cost-efficient marker-assisted selection in tea plants [25]. Despite the development of numerous tea tree markers, the growing complexity of studies necessitates more polymorphic and stable markers. ILP, a novel marker, remains relatively underutilized in the context of tea plants. In this study, a substantial number of ILP molecular markers of tea trees were developed based on the genomes of various tea varieties, including ‘Shuchazao’, ‘Longjing 43’, and the ancient tea tree DASZ [9,26,27,28,29,30]. These markers have shown cross-transferability through different species and can be used for identifying tea germplasm resources, analyzing genetic diversity, constructing linkage maps, QTL mapping tea plant importance traits, and molecular marker-assisted breeding.

## 2. Results

### 2.1. Tea ILP Marker Development

#### 2.1.1. Number, Distribution, and Density of ILP Markers in Tea Trees

**Figure 1:**
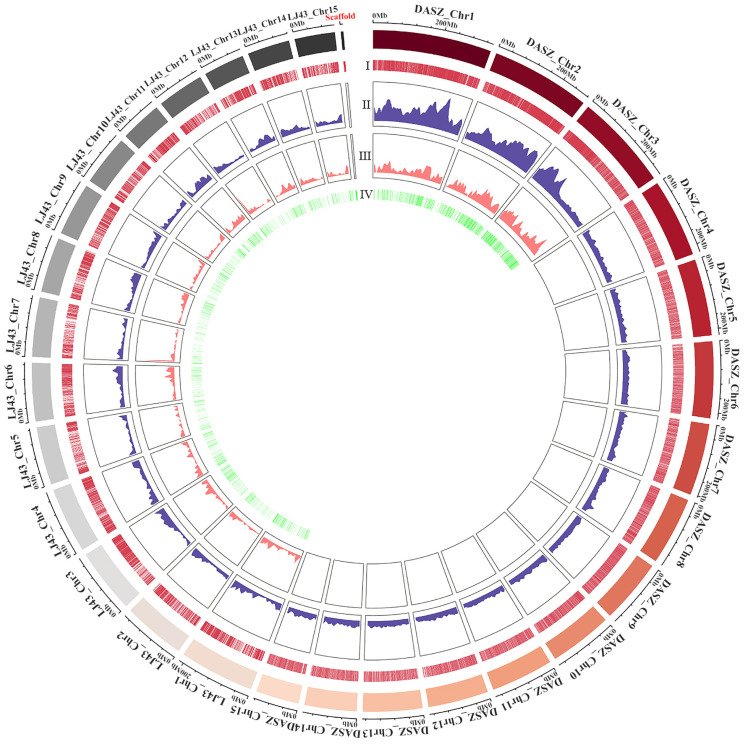
Circle diagrams illustrating the distribution and density of ILP primers developed based on the ‘Shuchazao’ scaffold genome, ‘Longjing 43’ chromosome-level genome, and DASZ chromosome-level genome, respectively. The outermost circle denotes the physical size (Mb) of 1 largest scaffold of ‘Shuchazao’, 15 chromosomes of ‘Longjing 43’ and 15 chromosomes of DASZ, each indicated by different colors. Circles I and II show the distribution positions and densities of designed ILP primers, respectively. Circles III and IV show the distribution density and location of developed ILP Makers, respectively.

A distribution density circle map was generated based on the position and size information of introns developed at first from the ‘Shuchazao’ scaffold genome, then from the ‘Longjing 43’ chromosome genome, and finally from the DASZ chromosome genome. The intron distribution density of the ‘Shuchazao’ scaffold genome was specifically constructed based on its largest scaffold (7.31 Mb) (Figure 1). A total of 206,886 pairs of ILP primers for tea plants were designed based on these three genomes. For the ‘Shuchazao’ scaffold genome, 105,127 primer pairs were designed. Among these, 53,016 primer pairs exhibited a single band through electronic PCR (ePCR) were distributed throughout 2895 scaffolds. The intron sequences varied in length, with the longest being 350 bp, the shortest 80 bp, and an average intron length of 262.39 bp. For the ‘Longjing 43’ chromosome-level genome, 48,232 primer pairs were designed, of which 33,815 primer pairs exhibited a single band based on ePCR. Out of these, 30,160 primer pairs were distributed to its 15 chromosomes with an average density of 13.05 primers/Mb. Primers were predominantly concentrated on both arms of the chromosomes, with the longest and shortest intron sequence lengths being 399 bp and 82 bp, respectively, and an average intron length of 279.67 bp. For the DASZ genome, a total of 53,527 primer pairs were designed, with 34,950 primer pairs showing a single band based on ePCR. Out of these, 34,915 primer pairs could be localized to its 15 chromosomes with an average density of 15.11 primers/Mb. The intron markers were most densely distributed on chromosomes 1, 2, and 3. The longest and shortest intron sequence lengths were 500 bp and 80 bp, respectively, with an average intron length of 266.84 bp. Using the designed primers and tea tree gene sequence information for ePCR, a total of 3214 tea ILP markers were ultimately developed. The circos graph displayed that 1393 markers developed from the Longjing 43 chromosome were evenly distributed through its 15 chromosomes, while 1342 markers developed from the tea tree DASZ chromosome were entirely distributed on chromosomes 1, 2, and 3 of ancient tea trees. The distribution of markers was relatively uniform. Chromosome DASZ_Chr3 had the most polymorphic markers, totaling 475, while LJ_Chr14 had the fewest polymorphic markers, totaling 65.

#### 2.1.2. Differential Distribution of Intron Length of Tea ILP

**Figure 2:**
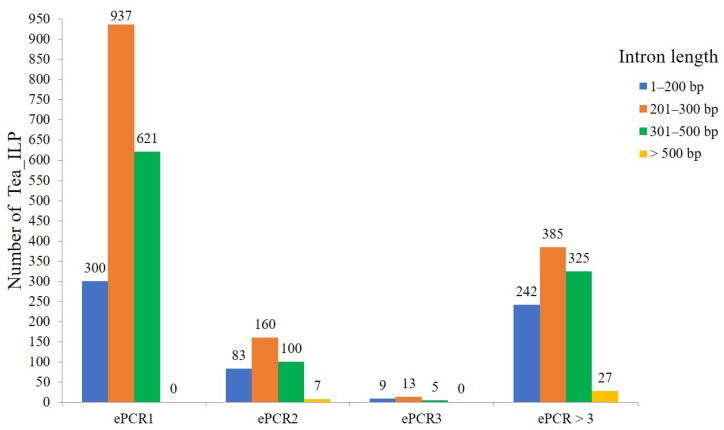
Distribution of intron length difference under different ePCR analysis. ePCR1 means 1 band amplified by ePCR, ePCR2 means 2 bands amplified by ePCR, ePCR3 means 3 bands amplified by ePCR, and ePCR > 3 means more than 3 bands amplified by ePCR.

After ePCR analysis, a total of 3214 tea ILP markers (Table S1) were developed through the three tea tree genomes. Among these markers, 1858 primer pairs produced a single band, 350 primer pairs generated two bands, 27 primer pairs yielded three bands, and 979 primer pairs resulted in more than three bands (Figure 2). Notably, there were 1495 introns with lengths ranging between 201–300 bp, followed by 1051 introns with lengths of 301–500 bp, and 634 introns of 1–200 bp. The number of introns with lengths greater than 500 bp was the lowest, with only 34.

#### 2.1.3. Experimental Validation of the Tea ILP Marker

Six tea tree species were subjected to PCR amplification using 230 selected tea ILP markers (Table 1 and Table S2). Among these, 213 pairs of primers successfully amplified products in all six species, with 112 primer pairs exhibiting polymorphism. The tea ILP markers displayed varying amplification efficiency and polymorphism rates through the six tea tree varieties. For ILP markers developed based on ‘Shuchazao’ scaffold genome, 39 pairs of ILP primer were synthesized, with 35 primer pairs achieving full amplification. Among these, 9 primer pairs exhibited polymorphism, resulting in a polymorphism rate of at least 23%. For ILP markers developed based on a ‘Longjing 43’ chromosome genome, 91 pairs of primers were synthesized, and 87 primer pairs achieved full amplification. Out of these, 38 primer pairs exhibited polymorphism, resulting in a higher polymorphism rate of about 42%. For ILP markers developed based on DASZ chromosome genome, 100 pairs of primer were synthesized, and 91 primer pairs achieved full amplification. Among these, 65 primer pairs exhibited polymorphism, resulting in the highest polymorphism rate of 65%. The polymorphism rate of primer pairs increased with the improvement in the reference genome assembly quality. Notably, the ILP markers developed entirely based on chromosomes exhibited the highest percentage of polymorphism.

### 2.2. Cross-Transferability of Tea ILP Markers among Different Plant Species

#### 2.2.1. Analysis of the Cross-Transferability of Tea ILP Markers among 11 Plant Species

**Figure 3:**
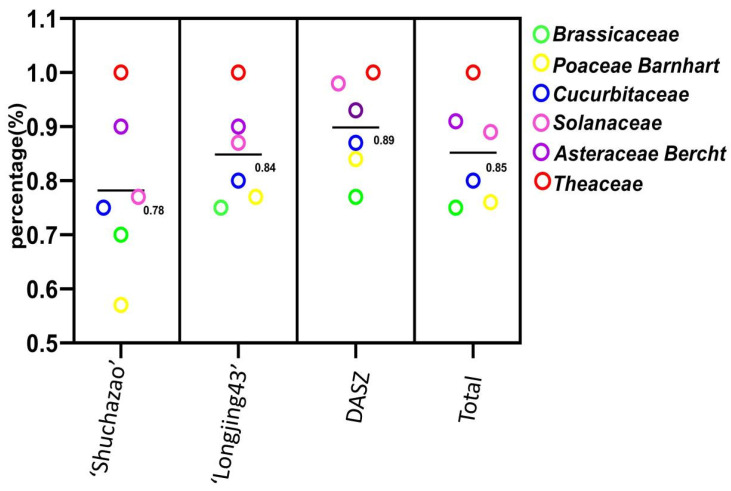
The cross-transferability of tea ILP markers in 6 families based on different reference genomes.

Figure 3 illustrates that 35 primer pairs (Table S3) had the ability to be transferred across 11 plant species of 6 families, with an average commonality amplification ratio of 0.85. All 35 tea ILP markers showed a 100% cross-transferability rate in *C. reticulata*. Tobacco, sunflower, wheat, tomato, cucumber, oilseed rape, and pepper all had a cross-transferability rate greater than or equal to 80%, while rice, *Arabidopsis thaliana*, and maize all had a cross-transferability rate of more than 60%. The tea ILP markers also demonstrated varying cross-transferability rates through the six families. Theaceae had the highest cross-transferability rate at 100%, followed by Asteraceae Bercht with 91.43%. Solanaceae, Cucurbitaceae, and Poaceae also displayed high cross-transferability rates, at 88.57%, 80.00%, and 76.19%, respectively. The cross-transferability rate was lowest in Brassicaceae, with 75.71%. The average cross-transferability rate of Tea ILP markers through the six families was 85.17%. Furthermore, the markers developed using the three different approaches exhibited transferability among the 11 species, with an average transferability rate of 89.83% for markers developed based on the DASZ chromosome genome of the ancient tea tree, 84.83% for markers developed based on the ‘Longjing 43′ chromosome genome, and 78.17% for markers developed based on the scaffold genome of ‘Shuchazao’.

#### 2.2.2. Analysis of Genetic Relationships in 11 Plant Species by Tea ILP Markers

**Figure 4:**
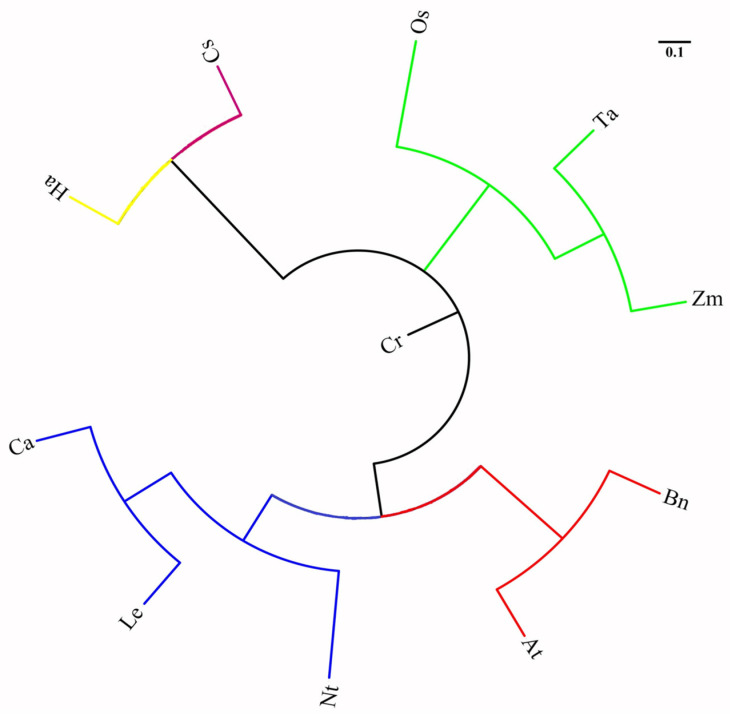
Genetic relationships of 11 plant species as determined by 35 ILP molecular markers. Cr (C. reticulate), Os (Oryza sativa), Ta (Triticum aestivum), Zm (Zea mays), Nt (Nicotiana tabacum), Le (Lycopersicon esculentum), Ca (Capsicum annuum), At (*A*. *thaliana*), Bn (*Brasscia napus*), Ha (*Helianthus annus*), Cs (*Cucumis sativus*).

Figure 4 illustrates the results of the NJ clustering of 11 plant species utilizing the 35 primer pairs of tea ILP (Table S3). The findings suggested that plants belonging to the same family exhibited a greater genetic similarity when clustered. For instance, Arabidopsis and oilseed rape of the Cruciferae clustered together, with a genetic similarity coefficient of 0.625. Similarly, wheat, maize, and rice of the Gramineae clustered together, showing a genetic similarity coefficient of 0.704. Furthermore, tomato, pepper, and tobacco of the Solanaceae formed a cluster with a genetic similarity coefficient of 0.674. Sunflowers of Asteraceae and cucumbers of Cucurbitaceae were positioned in separate branches from other families.

### 2.3. Genetic Diversity and Population Structure of C. tetracocca by 40 ILP Molecular Markers

#### 2.3.1. Genetic Diversity and Genetic Differentiation Analysis of Cultivated *C. tetracoccain* Puan

Genetic diversity analysis was conducted by 40 ILP molecular markers on 176 cultivated *C. tetracocca* trees located in the Qingshan, Louxia, and Digua towns of Puan County (Table 2). The results showed that a total of 169 observed alleles (Na) were identified. Among them, Tea_ILP900, Tea_ILP1097, Tea_ILP072, Tea_ILP290, and Tea_ILP2551 exhibited a minimum of 2 observed alleles (Na), while Tea_ILP1986 had a maximum of 10 Na. The average Na was 4.23. The number of effective alleles (Ne) varied from 1.31 to 7.63, with the smallest value observed at locus Tea_ILP2142 and the largest at locus Tea_ILP1986. The mean value of Ne was 2.58. Observed homozygosity (Obs-Ho) ranged from 0.01 to 1.00, with an average value of 0.78. On the other hand, expected homozygosity (Exp-Ho) ranged from 0.13 to 0.87, with an average value of 0.47. Observed heterozygosity (Obs-He) varied from 0.00 to 0.99, with a mean of 0.22, while expected heterozygosity (Exp-He) ranged from 0.13 to 0.87, with an average value of 0.53. The analysis revealed that observed heterozygosity was lower than expected heterozygosity. Shannon information index (I) values ranged from 0.27 to 2.11, with Tea_ILP3087 having the smallest and Tea_ILP1986 having the largest, with a mean value of 0.98. Nei gene diversity index (H) values ranged from 0.13 to 0.87, with Tea_ILP3087 having the smallest and Tea_ILP1986 having the largest, with a mean value of 0.53. Polymorphic information content (PIC) ranged from 12.10% to 85.48%. Among all loci, PIC values of Tea_ILP900, Tea_ILP1073, Tea_ILP2142, and Tea_ILP3087 were below 25%, indicating low polymorphism, while the remaining loci were all moderately and highly polymorphic, with a mean value of 47.56%.

Genetic analysis characterized cultivated *C. tetracocca* populations from three different regions in Puan, namely Qingshan Town, Louxia Town, and Digua Town (Table 3). The observed allele numbers (Na) were 4.10, 3.13, and 3.68, respectively. The effective allele numbers (Ne) were 2.57, 2.25, and 2.43, respectively, and the percentages of polymorphic loci (PPB) were 100% for all regions. The Shannon information indexes (I) were 1.01, 0.84, and 0.90, respectively, while the Nei gene diversity indexes (H) were 0.55, 0.48, and 0.50, respectively. The results indicated that the cultivated *C. tetracocca* population in Qingshan Town has higher genetic diversity and greater variability than the populations in the other two regions.

The inbreeding coefficient (*F*_is_) of Puan-cultivated *C. tetracocca* tree populations is 0.56, while the total population inbreeding coefficient (*F*_it_) is 0.57. Gene flow (*N*_m_) is estimated at 5.47, and the population differentiation coefficient (*F*_st_) is 0.04, indicating that 4.00% of the variation occurs between populations and 96.00% within populations (Table 4). An AMOVA analysis revealed that 6.40% of the total variation is due to inter-population differences, while 93.60% is due to intra-population variation, suggesting a high genetic similarity within the population (Table 4).

#### 2.3.2. Genetic Relationship and Genetic Structure of Cultivated *C. tetracocca* Population in Puan

Genetic similarity and distance were calculated in the three populations under study (Table 5). The genetic similarity coefficient ranged from 0.92 to 0.94, while the genetic distance ranged from 0.06 to 0.09. The highest genetic similarity coefficient, reaching 0.94, was observed between the populations of Louxia Town and Digua Town, with the smallest genetic distance recorded at 0.06. Conversely, the genetic similarity coefficient between the populations of Qingshan Town and Louxia Town was 0.92, and the genetic distance between these two populations was the largest at 0.09. These findings suggest a relatively close relationship between the populations of Louxia Town and Digua Town.

**Figure 5:**
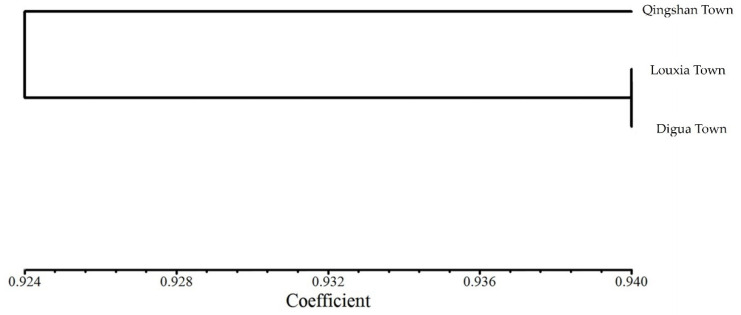
Population clustering map of Puan-cultivated *C. tetracocca* based on genetic similarity coefficient.

According to the clustering analysis conducted using NTsys 2.1, as illustrated in Figure 5, the cultivated *C. tetracocca* tree populations in Puan can be classified into two major groups when the threshold of genetic similarity coefficient is set at 0.924. The first group consists of cultivated *C. tetracocca* trees in Qingshan Town, while the second group encompasses trees in Louxia Town and Digua Town. This analysis validates the results obtained from the Popgene calculations, confirming the close relationship and high genetic similarity among the cultivated *C. tetracocca* trees in Louxia Town and Digua Town.

**Figure 6:**
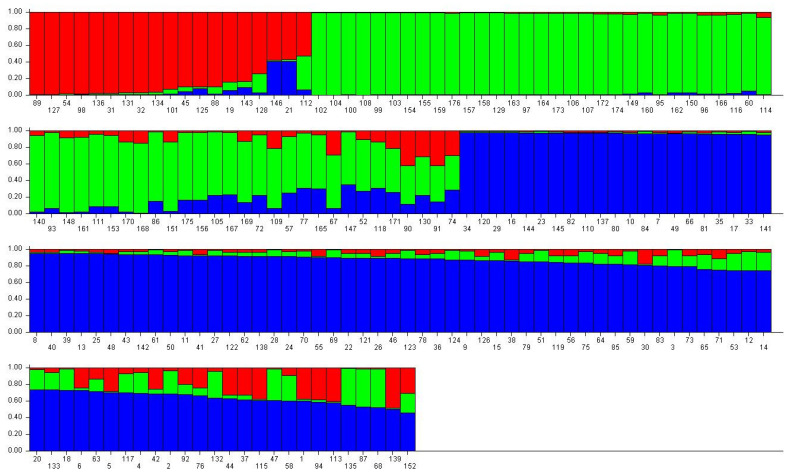
Q value distribution of population structure of cultivated *C. tetracocca* in Puan. The 176 individuals were divided into subpopulations S1 (red bar graph), S2 (green bar graph), and S3 (blue bar graph), comprising 19, 60, and 97 individuals.

Based on the analysis results, a value of K = 3, exhibiting the largest ΔK, was determined. Consequently, the number of subpopulations of *C. tetracocca* was identified as three, signifying that these groups have distinct genetic structures (Figure 6). The red bar graph represents the S1 group, which comprises 19 samples, with 13 originating from the population of Digua Town and 6 samples from the population of Qingshan. The green bar graph represents the S2 group, including 60 samples, with 53 tea trees originating from the population of Digua Town (88%), 6 tea trees from the population of Louxia Town (10%), and only 1 tea tree from the population of Qingshan (2%). The blue bar graph represents the S3 group, consisting of 97 samples, of which 48 tea trees belong to the population of Qingshan Town (accounting for 49%), 24 tea trees are from the population of Louxia Town (25%), and the remaining 25 tea trees come from the population of Digua Town (26%).

**Figure 7:**
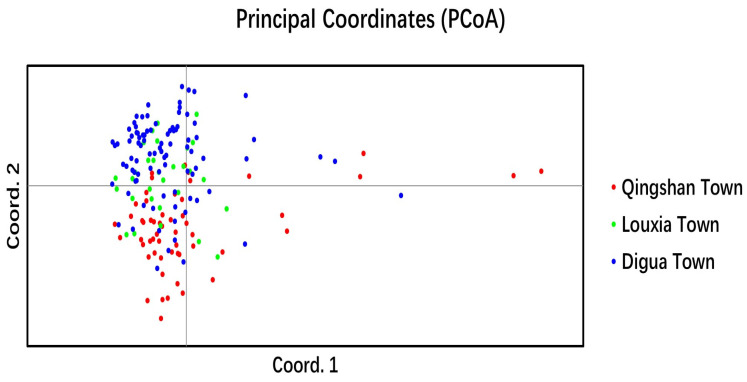
Principal component analysis of cultivated *C. tetracocca* in Puan.

Figure 7 displays the outcomes of the PCoA (principal coordinate analysis) applied to cultivated *C. tetracocca* trees in Puan. The analysis unveiled an initial clustering of cultivated trees within each region, with overlapping clusters attributed to frequent gene exchange between them. In comparison to the cultivated tea tree populations in these three regions, the cultivated *C. tetracocca* populations in Louxia Town and Digua Town exhibited a closer genetic relationship. These findings align with the results obtained from the cluster analysis and STRUCTURE analysis.

## 3. Discussion

The utilization of molecular markers in tea plants gained prominence relatively late, with significant advancements occurring towards the close of the 20th century [31]. Early investigations on molecular markers in tea trees primarily concentrated on the genomic region of tea trees, encompassing RFLPs [32,33], SNPs [34], and SSRs [35]. Contrary to the initial perception of being non-coding DNA, introns were recognized for their vital roles in gene expression regulation [36]. It was widely acknowledged that introns undergo evolution at a faster pace than exons, harboring more diversity within their regions [37]. A previous study had shown that the average count of intron SNPs among eight rice varieties was 12.1 per 1000 bp, which was almost three times higher than that in exons, which stands at just 3.6 per 1000 bp [38]. Moreover, the ILP marker is the only one that can identify polymorphism in the genic region [39]. Li et al. [40] reported that ILP outperformed markers designed through traditional methods in generating polymorphisms. Among simple PCR-based markers, ILPs demonstrated gene specificity, high variability, environmental neutrality, and co-dominance, resulting in a high transferability rate through related species [18]. The advancement of whole-genome sequencing along with the availability of robust in silico tools can accelerate the development of low-cost, highly efficient gene-associated functional molecular markers for genotyping [10]. Therefore, by harnessing the advantage of publicly available genome sequences of tea species [9,15,26], we identified introns in the whole genome to exploit their length polymorphism as molecular markers in plants. In this study, we designed 3214 primer pairs of tea ILP within the introns of the tea tree. The genomic coverage of the primers, developed based on the genomic level of the early scaffold of ‘Shuchazao’, was 33.46 ILPs per megabase (ILPs/MB), 17.27 ILPs/MB at the chromosome level of DASZ in ancient tea trees, and 20.87 ILPs/MB at the chromosome level of Longjing 43. In comparison to other reports, the ILP primer genome coverage of the tea tree was smaller than rice (44.2 ILPs/Mb) [41] and *Cleistogenes songorica* (1733.29 ILPs/Mb) [42] and slightly larger than cotton (13.13 ILPs/Mb) [43]. This disparity may be attributed to various factors, including intron conservativeness, divergent gene database sizes, and distinct tools developed for ILP markers among different species.

High polymorphism in intron regions, together with higher conservation in primer binding exonic sites, made ILP superior markers for diversity as well as cross-species transferability studies [19,23]. Numerous studies have consistently demonstrated the broad applicability of ILP markers through diverse species [44]. In our study, 230 tea ILP markers (Table S2) were randomly selected for amplification validation in six species of *Camellia*. Among these, 213 markers successfully amplified products in all six species, with 112 primer pairs exhibiting polymorphism. The success rate of ILP primers in tea plants was found to be significantly higher as compared to previous reports in onion (60.00%) [23] and wheat (75.30%) [37]. Moreover, 35 selected tea ILP markers exhibited an impressive average cross-transferability amplification rate of 85.17% ranging from 62.86 to 100% throughout 11 species, including representatives from Theaceae, Asteraceae, Solanaceae, Cucurbitaceae, Poaceae, and Brassicaceae. The amplification success rate of ILP markers was higher than that of *Medicago sativa* (51%) [14] and *C. songorica* (55.77%) [42]. Remaining primers could not amplify, either due to large intronic regions that were non-amplifiable or mutations in primer binding sites [23]. Additionally, this may be attributed to the prediction of ILPs using the tea genome as a reference. Markers from the expressed part of the genome (exons) were comparatively conserved showing high rates of transferability through cross-species amplification in related species. Thus, these ILP markers are well suited for genetic studies in different species as well [33]. A phylogenetic tree, devoid of roots, revealed clustering relationships among the 11 plant species under study. Arabidopsis and oilseed rape in the Cruciferae, rice, maize, and wheat in the Gramineae, and tomato, pepper, and tobacco in the Solanaceae were positioned closely, indicating that the transferability success decreases as the evolutionary distance between the source and target species increases, as reported previously [18]. The current study further demonstrated that ILP markers from the expressed regions of exons exhibited relatively high conservation and transferability through species amplification [37]. This not only underscored the extent of syntenic relationships, but also validated the efficacy of these newly developed markers in these species.

Molecular markers have played a pivotal role in investigating the domestication origin and evolution of cultivated tea plants. Since the late 20th century, scholars from China, India, Japan, and other countries have analyzed the genetic diversity and structure of various cultivated tea plants through different regions using diverse molecular marker techniques [45,46]. The success of DNA fingerprinting applications relies heavily on various marker attributes, including polymorphic potential, reproducibility, and discrimination power [47]. ILP markers, designed based on exon sequences flanking at least one intron region, have gained significance. Introns, characterized by fewer evolutionary constraints than exons and likely to be selectively neutral [48], facilitate the identification of polymorphisms that are essential for analyzing genetic diversity and population genetic structure. In the current study, ILP markers were also employed to analyze the genetic diversity of tea trees, revealing substantial genetic polymorphisms in Puan *C. tetracocca* variety resources. When evaluating genetic diversity within a germplasm collection, the polymorphism information content (PIC) and allelic richness offer insights into the level of polymorphism. A PIC less than 0.25 indicates little polymorphism, 0.25 < PIC < 0.5 signifies moderate polymorphism, and PIC > 0.5 denotes high polymorphism [49]. In the present study, the PIC of 40 primer pairs of ILP primers in Puan-cultivated *C. tetracocca* ranged from 12.10% to 85.48%, with an average of 47.56%, indicating moderate genetic diversity. A total of 169 alleles were detected, averaging 4.23 per ILP marker. While the genetic diversity obtained in this investigation was similar to that reported by other molecular markers such as SRAP with 6.05 alleles per primer combination [50], SCoT with the PIC ranged from 0.57 to 0.92 [51], and SNP [6] with the PIC ranged from 0.03 to 0.38. For tea trees, comparing diversity levels among different investigations remains challenging due to variations in the number and types of markers and genotypes used. This suggests that certain markers may be more polymorphic and informative than others. The Shannon information index (I) ranged from 0.27 to 2.11, with an average value of 0.98, and the Nei gene diversity index (H) ranged from 0.13 to 0.87, averaging 0.53. Among the three populations studied, the Qingshan Town tea tree population exhibited a higher genetic diversity than the other two populations. The cultivated tea trees in Qingshan Town clustered into a single taxon, while the cultivated tea trees in Louxia and Digua Towns clustered together due to higher genetic similarity. These results signify rich genetic variation in *C. tetracocca* tea trees, and ILP markers, as a novel molecular marker type, possess characteristics that are superior to other markers, enhancing the accuracy and efficiency of tea tree detection. They can be effectively utilized for screening and identifying existing tea tree varieties and breeding materials, providing significant support for breeding efforts.

## 4. Materials and Methods

### 4.1. Plant Materials and DNA Extraction

Six different species from the *Camellia* genus were used to ascertain the polymorphisms of ILP markers, including *C. reticulata*, *C. japonica*, *C. taliensis*, *C. sasanqua*, *C. nitidissima*, and *C. tetracocca*. Among them, *C. reticulate*, *C. taliensis*, and *C. nitidissima* are all endemic species to China, and *C. nitidissima* is an endangered plant species in China. *C. reticulata*, *C. japonica*, *C. sasanqua*, and *C. nitidisima* have important ornamental value, while *C. taliensis* and *C*. *tetracocca* are mainly used for producing tea. Furthermore, cross-transferability was evaluated in 11 plant species from six different families, encompassing *C. reticulata* (Cr), rice (*Oryza sativa*, Os), wheat (*Triticum aestivum*, Ta), corn (*Zea mays*, Zm), tobacco (*Nicotiana tabacum*, Nt), tomato (*Lycopersicon esculentum*, Le), cayenne pepper (*Capsicum annuum*, Ca), *A. thaliana* (At), oilseed rape (*Brassica napus*, Bn), sunflower (*Helianthus annuus*, Ha), and cucumber (*Cucumis sativus*, Cs). For the assessment of genetic diversity and population structure in *C. tetracocca*, a total of 176 plants were collected from Puan County in Guizhou Province. The sample collection locations are detailed in Table 3. DNA extraction was performed using the CTAB extraction method [52]. Subsequently, the integrity and quality of the extracted DNA were evaluated through 1.0% agarose gel electrophoresis.

### 4.2. Source of Sequences

The tea genome data analyzed in this study were sourced from assembled tea genomes at two scaffold levels and six chromosome levels. Among them, the whole-genome data for ‘Shuchazao’ *(C. sinensis* var. sinensis) scaffold level (AHAU_CSS) were retrieved from the NCBI Genomes database (https://www.ncbi.nlm.nih.gov/assembly/GCA_004153795.1/, accessed on 11 February 2019), with the project number being PRJNA510226 [15]. The whole-genome data for ‘Yunkang No. 10’ (*C. sinensis* var. assamica) scaffold level wwas downloaded from the NCBI Sequence Read Archive Database under accession PRJNA381277 [27]. The whole-genome data (CSS_V1) for ‘Shuchazao’ (*C. sinensis* var. sinensis) at the chromosome level were obtained from the website https://github.com/JiedanChen/TeaGenomeData (accessed on 21 April 2020) [28]. The genomic data for ancient tea trees DAASZ, ‘Longjing 43’ *(C. sinensis* var. sinensis), ‘Biyun’ *(C. sinensis* var. sinensis), ‘Huangdan’ *(C. sinensis* var. sinensis), and ‘Tieguanyin’ (*C. sinensis*) at the chromosome level were all sourced from the BIG data center, with project numbers PRJCA001158 [26], PRJCA002071 [9], PRJCA003382 [29], PRJCA002039 [30], and PRJCA003090 [53].

### 4.3. ILP Marker Development and ePCR Analysis

The development of ILP markers for tea trees involves the use of the IPv2.0 program, independently developed by the Industrial Crops Research Institute, Yunnan Academy of Agricultural Sciences. The software has been registered with the copyright number 2021SR0437322. The basic process entails importing the annotation file and corresponding genome sequence file of the tea tree genome into the server and inputting the upper limit of intron length. Subsequently, the IPv2.0pl script is executed to retrieve intron information, including gene location, length, and sequence. The tea tree genome sequence information file is then uploaded, and the ILP primer design sequence is generated in the ILP_p3in.pl script, utilizing the intron information obtained in the previous step. Finally, the result file generated by the ILP_p3out script is employed to design tea tree ILP primers using Primer3.0. To further refine primer selection and maximize the utility of tea tree ILP primers, the designed ILP primers were screened using the ePCR method in silico [54] with the following parameters: 4 bp mismatch, 2 bp gap, 60 bp margin, and 80–1200 bp product size. The target tea tree ILP markers are subsequently screened based on the results of this ePCR analysis.

The markers developed for ILP are consistently named with the prefix Tea_ILP followed by a unique identifier, such as Tea_ILP0001. Using the annotation data from the ‘Shuchacao’ scaffold genome [15], primer pairs were designed to target introns with fragments smaller than 300 bp, then 105,127 primer pairs were obtained. Among them, a total of 23,948 pairs of primers showed one band in both the ‘Shuchacao’ scaffold genome and RNA databases through ePCR assay. Furthermore, 479 tea ILP markers were randomly selected and ePCR parameters were reset. Finally, 39 markers were selected by ePCR for subsequent experimental validation. Based on the annotation information of the ‘Longjing43’ chromosome genome [9], primer pairs were designed for introns with fragments less than 300 bp, and a total of 48,232 primer pairs were designed. ePCR analysis was conducted at the chromosome genome level of ‘Longjing 43’ [9], as well as the scaffold levels of ‘Yunkang 10’ [27] and ‘Shuchazao’ [15], then 1393 primer pairs of tea ILP markers with one band were selected. Finally, 91 pairs of primers were randomly selected from these 1393 primer pairs of tea ILP markers for synthesis and experimental validation. Based on the annotation information of the ancient tea tree DASZ chromosome genome, primer pairs were designed for introns with fragments less than 300 bp, and a total of 53,527 primers pairs were designed. ePCR analysis was performed at the chromosome genomic level of ‘Longjing 43’ [9], ‘Shuchazao’ [28], ‘Biyun’ [29], ‘Huangdan’ [30], and ‘Tieguanyin’ [53], and 1342 primer pairs of tea ILP markers showed differences through six genomes. Finally, 100 primer pairs were randomly selected from these 1342 primer pairs of tea ILP markers for synthesis and subsequent experimental verification (Table 6).

### 4.4. ILP Molecular Marker Detection

To validate the amplification and polymorphic efficiency of the tea ILP markers, 230 primer pairs were randomly selected from three different reference genome of tea plant. These markers exhibited an ILP size range of 200–300 bp according to the ePCR results. All primers were synthesized by Shanghai Bioengineering Technology Co., Ltd., and detailed information about the 230 primer pairs can be found in Table S2. PCR was performed in a 10 μL volume that contained 0.5 U of Taq DNA polymerase, 0.2 mM dNTP, 50 ng of DNA template, 0.5 μM each primer, and PCR buffer with 10 mM Tris PH 9.0, 50 mM KCl, and 1.5 mM MgCl_2_. DNA amplification was conducted with the following thermal profile: initial denaturation at 9 °C for 3 min; 13 cycles of 30 s at 94 °C, 45 s at 65 °C with 0.7 °C decrease in annealing temperature per cycle and 1 min at 72 °C; 23 cycles of 30 s at 94 °C, 45 s at 56 °C, and 1 min at 72 °C and a final extension at 72 °C for 5 min. The PCR products were separated on 6% denaturing polyacrylamide gels and were visualized by silver staining.

### 4.5. Cross-Transferability of Tea ILP

In each case, the primer and species transferability were calculated on the basis of successful amplifications of targeted markers loci in each species. Further, to explore the wider applications of the markers developed in the study, 35 pairs of primers were used to amplify the genomic DNA of six botanical families including 11 plant species. The primer pairs chosen for the cross-species evaluation are detailed in Table S3.

### 4.6. Genetic Diversity and Population Structure Analysis of C. tetracocca

We have chosen 40 primer pairs with abundant polymorphism and clear amplification bands from the initial set of 230 ILP primer pairs. The specifics of these 40 primer pairs are provided in Table S4.

### 4.7. Data Statistics and Analysis

We conducted an analysis of the number, distribution, and density of ILP markers developed based on different reference genomes. The distribution density circles were generated using the “cicros” tool on the OmicStudio platform, incorporating clustering information related to introns. Additionally, the distribution of intron length differences was mapped using GraphPad Prism 9 (Prism—GraphPad) (GraphPad Software, San Diego, CA, USA).

The electropherograms obtained after the experiments were manually examined and recorded. Clearly displayed bands were denoted as “1”, while those without bands were marked as “0”, creating a raw data set of 0 s and 1 s (Tables S5 and S6) in an Excel sheet. Then the raw data set of 0 s and 1 s was transformed into genotype data using DataFormatter 2.7 [55] software. We employed NTSYSpc-2.1 software [56] to compute genetic distances for all 11 species. The resulting genetic distance matrix was then imported into MEGA 7.0 software [57] to construct a rootless phylogenetic tree based on the neighbor-joining (NJ) method. Popgene 1.31 software [58] was then employed to analyze the genetic diversity of *C. tetracocca*, calculating the number of observed alleles (Na), effective allele number (Ne), Shannon index (I), observed homozygosity (Obs_Hom), observed heterozygosity (Obs_Het), expected homozygosity (Exp_Hom), expected heterozygosity (Exp_Het), Nei gene diversity index (H), genetic similarity coefficient (S), genetic distance (D), gene flow (Nm), and other relevant data. Based on the allele frequencies obtained from the analysis, the primer polymorphic information content (PIC) value was computed using PowerMarker 3.25 [59]. For the analysis of population genetic structure, the raw data were converted into the structure data format using DataFormatter 2.7 [57]. Subsequently, Structure 2.3.4 software was utilized to analyze the genetic structure of the population. The group structure calculation’s K value was set to 2~7, with each K value repeated 10 times. The burn-in period was set to 50,000, and MCMC was set to 100,000 iterations. The determination of the best value of K was based on the likelihood value LnP (D) and ΔK. Finally, the data were re-evaluated using PCoA with GenALEx 6.5 [60] software.

## 5. Conclusions

In the present study, 3214 pairs of tea ILP primers were ultimately developed through three different reference genomes using ePCR assay. Among them, 230 pairs of tea ILP primers were randomly selected for amplification across six *Camellia* species, 35 pairs of primers were used to assess the cross-transferability among 11 species in six families, and 40 pairs of primers were utilized to evaluate the genetic diversity and population structure of *C. tetracocca* in Puan County, Guizhou Province. The above indicates that the genome-wide tea ILP markers developed in this study with high cross-species transferability rate can be used not only for tea plant population genetics research but also for other species of the Camelliaceae with unknown genomic information or for species outside the Camelliaceae.

## Figures and Tables

**Table 1:** Statistics regarding amplification and polymorphic efficiency of 230 ILP primers developed based on three reference genomes in six tea tree species.

Origin of Primers	Primers Synthesis	Amplified Primers (%)	Polymorphic Primers (%)
‘Shuchazao’ [15]	39	35 (89.74)	9 (23.80)
‘Longjing 43’ [9]	91	87 (95.60)	38 (41.76)
DASZ [26]	100	91 (91.00)	65 (65.00)
Total	230	213 (92.61)	112 (48.70)

**Table 2:** Genetic diversity analysis of Puan-cultivated *C. tetracocca* based on ILP molecular markers.

Marker ID	Na	Ne	I	Obs-Ho	Obs-He	Exp-Ho	Exp-He	H	PIC%
Tea_ILP1116	6.00	3.89	1.53	1.00	0.00	0.26	0.74	0.74	70.71
Tea_ILP1418	4.00	2.13	0.83	0.01	0.99	0.47	0.53	0.53	41.91
Tea_ILP1396	4.00	2.16	0.92	0.81	0.19	0.46	0.54	0.54	46.58
Tea_ILP1000	5.00	3.29	1.31	1.00	0.00	0.30	0.70	0.70	63.95
Tea_ILP1589	3.00	1.87	0.71	0.34	0.66	0.53	0.47	0.46	37.12
Tea_ILP900	2.00	1.32	0.41	0.72	0.28	0.76	0.24	0.24	21.40
Tea_ILP1097	2.00	1.97	0.69	1.00	0.00	0.51	0.49	0.49	37.11
Tea_ILP1023	6.00	3.54	1.42	0.95	0.05	0.28	0.72	0.72	66.92
Tea_ILP1222	3.00	1.76	0.77	0.76	0.24	0.57	0.43	0.43	39.22
Tea_ILP1192	4.00	1.59	0.73	0.80	0.20	0.63	0.37	0.37	34.69
Tea_ILP1073	3.00	1.26	0.39	0.80	0.20	0.80	0.20	0.20	18.71
Tea_ILP591	7.00	5.00	1.72	1.00	0.00	0.20	0.80	0.80	77.16
Tea_ILP1158	4.00	3.86	1.37	0.47	0.53	0.26	0.74	0.74	69.26
Tea_ILP072	2.00	1.62	0.57	0.48	0.52	0.62	0.38	0.38	30.99
Tea_ILP015	4.00	3.07	1.16	1.00	0.00	0.32	0.68	0.67	60.45
Tea_ILP290	2.00	1.78	0.63	1.00	0.00	0.56	0.44	0.44	34.21
Tea_ILP380	5.00	3.74	1.40	0.53	0.47	0.27	0.74	0.73	68.66
Tea_ILP450	3.00	1.52	0.57	0.59	0.41	0.66	0.34	0.34	29.16
Tea_ILP202	3.00	2.48	0.98	1.00	0.00	0.40	0.60	0.60	51.77
Tea_ILP284	4.00	2.68	1.07	1.00	0.00	0.37	0.63	0.63	55.01
Tea_ILP1875	8.00	6.07	1.92	1.00	0.00	0.16	0.84	0.84	81.51
Tea_ILP1946	4.00	3.14	1.20	1.00	0.00	0.32	0.68	0.68	61.60
Tea_ILP1986	10.00	7.63	2.11	1.00	0.00	0.13	0.87	0.87	85.48
Tea_ILP2114	7.00	3.01	1.32	1.00	0.00	0.33	0.67	0.67	61.18
Tea_ILP2142	4.00	1.31	0.49	0.97	0.03	0.77	0.24	0.23	21.94
Tea_ILP2171	5.00	2.67	1.24	1.00	0.00	0.37	0.63	0.63	58.70
Tea_ILP1923	3.00	2.08	0.78	0.10	0.90	0.48	0.52	0.52	40.57
Tea_ILP1924	5.00	1.35	0.60	1.00	0.00	0.74	0.26	0.26	25.02
Tea_ILP1945	4.00	2.11	0.86	0.26	0.74	0.47	0.53	0.53	43.27
Tea_ILP1951	3.00	1.78	0.76	0.56	0.44	0.56	0.44	0.44	38.52
Tea_ILP1967	4.00	3.11	1.18	1.00	0.00	0.32	0.68	0.68	61.01
Tea_ILP1982	5.00	1.46	0.66	1.00	0.00	0.68	0.32	0.32	29.83
Tea_ILP1991	4.00	1.85	0.75	0.40	0.60	0.54	0.46	0.46	37.74
Tea_ILP2017	6.00	2.83	1.34	1.00	0.00	0.35	0.65	0.65	61.13
Tea_ILP2551	2.00	1.54	0.54	0.55	0.45	0.65	0.35	0.35	28.96
Tea_ILP3195	5.00	2.71	1.15	1.00	0.00	0.37	0.63	0.63	55.95
Tea_ILP1959	3.00	2.00	0.74	0.15	0.85	0.50	0.50	0.50	38.65
Tea_ILP3087	3.00	1.15	0.27	0.88	0.13	0.87	0.13	0.13	12.10
Tea_ILP1953	4.00	2.97	1.13	1.00	0.00	0.34	0.66	0.66	59.18
Tea_ILP2343	4.00	2.02	0.92	1.00	0.00	0.49	0.51	0.50	45.10
Mean	4.23	2.58	0.98	0.78	0.22	0.47	0.53	0.53	47.56

Note: number of observed alleles, Na; number of effective alleles, Ne; shannon information index, I; observed Homozygosity, Obs-Ho; observed heterozygosity, Obs-He; observed heterozygosity, Obs-He; expected Homozygosity, Exp-Ho; expected heterozygosity, Exp-He; Nei gene diversity index, H; polymorphic information content, PIC%.

**Table 3:** Analysis of genetic diversity among populations of Puan-cultivated *C. tetracocca* based on ILP molecular markers.

Population	Sample Size	Longitude	Latitude	Elevation (m)	Ground Diameter (cm)	Na	Ne	I	H	PPB%
Qingshan Town	55	104.96–104.97	25.42–25.43	1697–1721	18–39	4.1	2.57	1.01	0.55	100
Louxia Town	30	104.98–104.99	25.40–25.42	1574–1588	23–44	3.13	2.25	0.84	0.48	100
Digua Town	91	104.98–104.99	25.75–25.76	1675–1875	16–28	3.68	2.43	0.9	0.5	100

Note: number of observed alleles, Na; number of effective alleles, Ne; Shannon information index, I; Nei gene diversity index, H; percentages of polymorphic loci, PPB%.

**Table 4:** Genetic differentiation among populations of cultivated *C. tetracocca* in Puan.

Popgene				AMOVA				
*F*_is_	*F*_it_	*F*_st_	*N*_m_	Source of Variation	df	SS	Var. Components	PMV (%)
0.56	0.57	0.04	5.47	Among populations	2	183.68	1.35	6.40%
				Within populations	173	3419.25	19.76	93.60%

Note: inbreeding coefficient within a population *F*_is_; total population inbreeding coefficient, *F*_it_; population differentiation coefficients, *F*_st_; population gene flow, *N*_m_; degrees of freedom, df; square deviation, SS; variance components, var. components; percentage of molecular variance, PMV.

**Table 5:** Analysis of genetic similarity and genetic distance among populations of Puan-cultivated *C. tetracocca* based on ILP markers.

Population	Qingshan Town	Louxia Town	Digua Town
Qingshan Town	1.00	0.92	0.93
Louxia Town	0.09	1.00	0.94
Digua Town	0.07	0.06	1.00

Note: The upper right part is the genetic similarity coefficient, and the lower left part is the genetic distance.

**Table 6:** Tea ILP molecular markers developed based on three different reference genomes.

References	Number of Designed Primers	Number of Primers Used in ePCR Analysis	Number of Synthetized Primers
‘Shuchazao’ [15]	105,127	479	39
‘Longjing 43′ [9]	48,232	1393	91
DSAZ [26]	53,527	1342	100

## Data Availability

All data were shown in Tables and Figures in the main text or Supplementary Materials.

## Supplementary Materials

The following supporting information can be downloaded at: https://www.mdpi.com/article/10.3390/ijms25063241/s1.
